# Diets and Joint Symptoms: A Survey of Moroccan Patients With Chronic Inflammatory Rheumatic Disease

**DOI:** 10.7759/cureus.53868

**Published:** 2024-02-08

**Authors:** Nihad Takhrifa, Fatima Zahrae Taik, Imane Berrichi, Anass Adnine, Fatima Ezzahra Abourazzak

**Affiliations:** 1 Rheumatology, Mohammed VI University Hospital Center of Tangier, Tangier, MAR

**Keywords:** joint symptoms, spondyloarthritis, rheumatoid arthritis, chronic inflammatory rheumatic disease, diet

## Abstract

Introduction

The role of diet in the onset or aggravation of chronic diseases, especially chronic inflammatory rheumatic disease (CIRD), such as rheumatoid arthritis (RA) or spondyloarthritis (SpA), is a question frequently asked by patients. Our study aims to investigate whether Moroccan patients report a relationship between certain diets and disease symptoms and to study whether patients adopt specific dietary behaviours in order to relieve their symptoms.

Methods

This is a cross-sectional survey that included all patients followed for CIRD. The questionnaire has three parts, patients' sociodemographic and clinical data, patients' beliefs and attitudes regarding diet in relation to their joint symptoms, and a list of 24 foods for which patients were asked to indicate whether they aggravate, improve, or leave their joint symptoms unchanged.

Results

Thirty-four percent of the patients reported that the food had an effect on their symptoms, with 25% of them reporting an aggravation. Honey, garlic, and olive oil were the foods most often reported to improve joint symptoms, while red meat, fish, and legumes were most often reported to worsen symptoms. Age and type of rheumatism were factors associated with reporting that food affects joint symptoms. Twenty-three percent of the patients stated that they had already had discussions about diet with their rheumatologists, while 85.7% showed interest in such discussions. Experience with a food that improves joint symptoms was the only factor associated with discussing the diet with a rheumatologist.

Conclusion

Nearly one-third of the patients with CIRD reported an effect of diet on their joint symptoms.

## Introduction

Food is a major environmental factor in human health [1]. It is a current topic and a fairly frequent preoccupation of patients. Chronic diseases such as obesity, diabetes, atherosclerosis, osteoporosis, and various cancers can be considered as nutrition- or environment-related diseases. Different studies indicate that specific foods or diets are able to influence the development and outcome of these chronic diseases. The majority of chronic diseases (e.g., obesity, diabetes) are strongly influenced by nutrition, and food metabolism is closely related to inflammatory processes [2]. However, long-term prospective clinical trials have not yet been completed to confirm the described correlation between diet and chronic disease [3]. The question of whether diet plays a role in the development of chronic inflammatory rheumatic disease (CIRD), such as rheumatoid arthritis (RA) or spondyloarthritis (SpA), or can affect the course of the disease is an important issue for many patients and healthcare providers, and the results are controversial [4].

According to the literature, diet seems to play an important modulating role in CIRD. Diet is considered an environmental factor, which can have effects on inflammation, antigen presentation, antioxidant defense mechanisms, and gut microbiota [5]. CIRD is a group of chronic diseases that most often requires long-term immunomodulatory therapies to suppress joint inflammation, and despite advances in the treatment of these diseases and the development of biologic therapy, which has revolutionised the treatment of SpA and RA, remission rates are still low [6], which may be associated with poor compliance leading patients to search for non-drug treatment options such as certain diets and dietary practices. In recent years, an increasing number of studies have investigated the role of diet and nutrition as potential tools for the prevention and management of RA [7].

Patients with chronic diseases, including CIRD, often ask about the benefits of diet in relieving their joint symptoms. Some of them have already tried a diet or had an experience with a food that made their symptoms worse or better. However, the associations found between nutrition and these diseases are often weak and contradictory, and clinical trials on nutrition suffer from methodological flaws [1].

The Moroccan population is increasingly expressing greater interest in dietary choices, particularly influenced by the media. However, the challenge lies in the limited scientific evidence regarding their effectiveness and the absence of clear recommendations. As a result, many patients undertake various dietary interventions.

The primary objective of this study is to investigate whether Moroccan patients report a relationship between certain diets or foods and joint symptoms and, as secondary objectives, to investigate associated factors including adherence to therapy and whether CIRD patients have discussions with their rheumatologists about diet.

## Materials and methods

Study design

This is a cross-sectional study, conducted between February 2021 and November 2022, including all patients followed up for CIRD who present to the Rheumatology Department of the Mohammed VI University Hospital Center of Tangier.

This study was approved by the Ethics Committee of the Hospital-University of Tangier (CEHUT) (approval number: 01/2022), and a signed informed consent was obtained from all patients.

Population

In this survey, patients followed for CIRD were recruited. Subjects were included if they met the 1987 American College of Rheumatology or American College of Radiology (ACR)/European Alliance of Associations for Rheumatology (EULAR) 2010 criteria for RA and the Assessment of Spondyloarthritis International Society (ASAS) criteria for SpA, had been followed for more than six months for their rheumatic disease, and were older than 18 years. Patients with psychiatric disorders and those who refused to participate in the study were excluded. All patients declared their consent before entering the study.

Questionnaire

In this study, the evaluation of the patients' beliefs and attitudes regarding diet was carried out by using a questionnaire (see Appendices), which was elaborated by two professors of rheumatology, who are among the authors (FZT and FEA). The questionnaire was based on previous studies [8,9] and adapted to the Moroccan context. A cognitive interview was then carried out with five patients who were asked to give their opinion on each element of the questionnaire (acceptability and clarity) and to indicate if there was any item that could be added or removed. Some changes were made thereafter. Afterwards, a committee (three rheumatologists and one methodologist) reviewed the final version of the questionnaire and approved its content. Finally, we conducted a pilot study with a group of 30 patients to examine the acceptability and usability of the questionnaire and to identify possible misunderstandings and misinterpretations. All participants stated that there was no ambiguity in any item of the questionnaire. Test and retest reliability for single-item correlations were strong and statistically significant (p<0.001). Finally, we performed a logistic regression that showed the results we expected demonstrating an adequate construct validity of the questionnaire.

This questionnaire consists of three parts. The first part included sociodemographic data, comorbidities, and information on CIRD including type, duration of disease, disease activity (Disease Activity Score in 28 Joints (DAS28) for RA and ASDAS for SpA), functional impact (Health Assessment Questionnaire for RA and Bath Ankylosing Spondylitis Functional Index for SpA), and current treatments. The second part consisted of 12 closed-ended questions with a variety of sub-questions, both closed and open. The questions were aimed at determining whether patients believe diet influences rheumatic activity, their experiences with foods that worsen or improve joint symptoms, and whether they avoid certain foods that exacerbate symptoms or adopt specific diets to alleviate joint symptoms. Additionally, the survey examined the influence of fasting, the use of dietary supplements, and patients' discussions with their rheumatologists regarding diet. The third part consisted of a list of 24 foods (garlic, olive oil, argan oil, saffron, honey, black cumin, fish, ginger, curcumin, cloves, fenugreek, red meat, eggs, tea, coffee, tomatoes, pomegranates, soda, chocolate, milk, yoghurt, dates, dried fruit, legumes), for which patients were asked to indicate whether they aggravate, improve, or leave their joint symptoms unchanged.

Thus, patient adherence was assessed by a self-administered Compliance Questionnaire for Rheumatology (CQR), with a validated threshold of 80%. A score of 80% or more places the patient in the "good adherence" category, and a lower score places the patient in the "patients with adherence problems" category.

Statistical analysis

The data were entered and analysed using IBM SPSS Statistics for Windows, Version 21.0 (Released 2012; IBM Corp., Armonk, New York, United States). A descriptive analysis was done, and the qualitative variables were expressed in numbers and percentages and the quantitative variables in means and standard deviations or medians and quartiles according to the distribution of the variable. Then, a univariate/multivariate analysis was performed to study the association between sociodemographic and clinical factors and reporting that diet affects joint symptoms and to study patients' discussions with their rheumatologists about diet and associated factors.

## Results

Demographic and clinical characteristics of patients

We have included 168 patients. The characteristics of the patients who responded to the survey are presented in Table 1.

**Table 1:** Demographic and clinical characteristics of study patients *: data presented as mean±SD; **: data presented as a percentage (%); ***: data presented as median VAS: Visual Analogue Scale; cDMARDs: conventional synthetic disease-modifying antirheumatic drugs; RA: rheumatoid arthritis; SpA: spondyloarthritis; CQR: Compliance Questionnaire for Rheumatology

Parameters	N=168
Age, years* (±SD)	46.37±13.6
Female gender (%)**	74.4%
Comorbidity (%)**	34.5%
Low level of education (%)**	54.2%
RA (%)**	61.9%
SpA (%)**	38.1%
Disease duration (years)^***^	7 years (3-14.7)
VAS pain* (±SD)	4.7±1.5
Disease activity: remission (%)**	21.4%
Disease activity: high activity (%)**	78.6%
No treatment (%)**	6.5%
cDMARDs (%)**	71.4%
Glucocorticoids (%)**	48.8%
Biologics (%)**	3%
CQR mean* (±SD)	53.4±9.5
Good compliance with treatment (%)**	58.3%

The average age was 46.37±13.6, 74.4% of the patients were women, 34.5% had comorbidities, 54.2% were illiterate, and 61.9% were followed for RA and 38.1% for SpA. The median disease duration was seven years (3-14), and the mean Visual Analogue Scale (VAS) pain was 4.7±1.5. Three-quarters of the patients were in a disease flare with very high activity, and half of the patients reported a functional impact of the disease. With regard to treatment, 71.4% of the patients in our study were on conventional synthetic disease-modifying antirheumatic drugs (cDMARDs), almost half (48.8%) of the patients were taking corticosteroids, and only 3% of the patients were using biological disease-modifying antirheumatic drugs (bDMARDs), while 6.5% were not receiving any treatment. The mean CQR score was 53.4±9.5. Around 58.3% of the patients had good compliance.

Impact of diet on CIRD joint symptoms

Thirty-four percent of the patients reported that food had an effect on their CIRD, with 25% of them reporting an aggravation and 9.5% an improvement. Seventeen percent of the patients reported food avoidance behaviours, while 6% adopted certain diets, and 3% had tried fasting to relieve joint symptoms. Fourteen percent of the patients used food supplements (13.7% vitamin D), and one-third of these patients reported an improvement (Table 2).

**Table 2:** Patients' beliefs and attitudes on the relationship between diet and joint symptoms CIRD: chronic inflammatory rheumatic disease

Items	N=168
Patients report that food had an effect on their CIRD	34.5%
Patients report that food worsened their symptoms	25%
Patients report that food improved their symptoms	9.5%
Patients report avoidance of certain foods	17.9%
Patients adopt certain diets to relieve joint symptoms	6%
Patients have tried fasting to relieve joint symptoms	3%
Patients use food supplements	14.4%
Patients use vitamin D supplements	13.7%
Patients reported that they have already had discussions about diet with their rheumatologists	23.2%
The reason why patients never discussed the diet with their rheumatologists is because the doctor never mentioned it	99.2%
Patients are willing to discuss diet with their rheumatologists	85.7%

**Figure 1:**
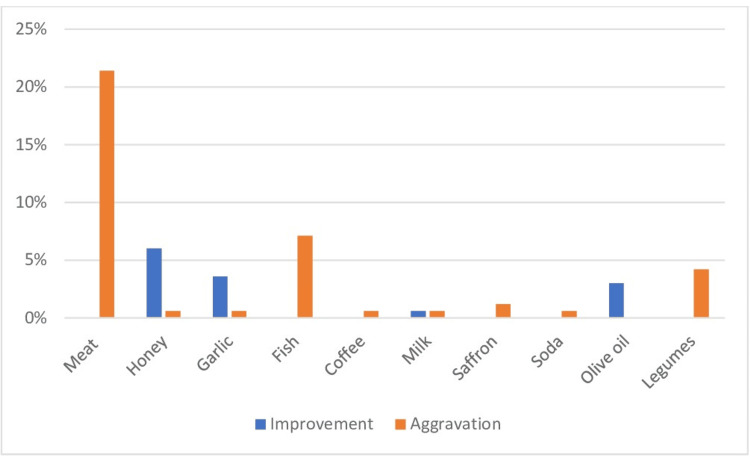
Foods reported to aggravate or improve joint symptoms

Honey (6%), garlic (3.6%), and olive oil (3%) were the foods most often reported to improve CIRD symptoms, while red meat (21.4%), fish (7.1%), and legumes (4.2%) were the most often reported to worsen symptoms (Figure 1).

In univariate and multivariate analysis, age (OR: 1.001; CI 95% (1.003-1.024); p=0.015) and type of rheumatism (RA vs SpA) (OR: 2.688; CI 95% (1.309-5.520); p=0.007) were the factors associated with reporting that food affects joint symptoms. On the contrary, gender, comorbidities, duration of disease, VAS pain, functional impact, and therapeutic adherence were not associated (Table 3).

**Table 3:** Univariate and multivariate analysis for the influence of diet on joint symptoms *: p-value significant at <0.05; P: probability of error; RA: rheumatoid arthritis; SpA: spondyloarthritis; VAS: Visual Analogue Scale; CQR: Compliance Questionnaire for Rheumatology

Parameters	Univariate analysis		Multivariate analysis	
	OR (CI 95%)	P-value	OR (CI 95%)	P-value
Age (years)	0.974 (0.949-0.999)	p=0.038	1.001 (1.003-1.024)	P=0.015^*^
Gender	0.486 (0.202-1.142)	p=0.098	-	-
Comorbidity	1.229 (0.601-2.512)	p=0.573	-	-
Level of education	1.343 (0.941-1.918)	p=0.104	-	-
RA vs SpA	2.294 (1.088-4.839)	p=0.029	2.688 (1.309-5.520)	P=0.007^*^
Duration of disease	0.970 (0.932-1.009)	p=0.127	-	-
VAS pain	1.039 (0.834-1.294)	p=0.731	-	-
Disease activity	1.333 (0.604-2.943)	p=0.476	-	-
Functional impact	0.473 (0.240-0.932)	p=0.031	0.711 (0.356-1.421)	P=0.335
CQR	1.033 (0.998-1.069)	p=0.067	-	-

Patients' discussions with their rheumatologists about diet

Twenty-three percent of the patients stated that they had already had discussions about diet with their rheumatologists, while 85.7% showed interest in such discussions. For those who had never done so, the main reason was that the rheumatologist had never brought up the subject (Table 2).

In univariate and multivariate analysis, discussion of the diet was associated with the experience of consuming a food that improved joint symptoms (OR: 3.426; CI 95% (1.379-8.514); p=0.008) (Table 4).

**Table 4:** Univariate and multivariate analysis for patients' discussion of diet with their rheumatologists *: p-value significant at <0.05; P: probability of error; VAS: Visual Analogue Scale

Parameters	Univariate analysis		Multivariate analysis	
	OR (CI 95%)	P-value	OR (CI 95%)	P-value
Experience with a food that improves symptoms	0.903 (1.357-11.226)	p=0.012	3.426 (1.379-8.514)	p=0.008^*^
Duration of disease	1.084 (1.049-1.121)	P<0.001	1.005 (0.961-1.052)	p=0.823
VAS pain	1.269 (1.178-1.367)	P<0.001	0.943 (0.738-1.206)	p= 0.642
Functional impact	2.833 (1.656-4.849)	P<0.001	0.701 (0.310-1.558)	p=0.383

## Discussion

The main objective of the study was to study the influence of diet on joint symptoms in patients with CIRD.

One-third of the patients reported that food influenced their symptoms, with 25% of them reporting an aggravation. Among a list of 24 specific foods, some foods were most often reported to improve joint symptoms, while others were reported to make joint symptoms worse. The reporting of food affecting joint symptoms was only associated with an individual's age and the type of rheumatism. Nearly one-quarter of the patients stated that they had already had discussions about diet with their rheumatologists, and experience with a food that improves joint symptoms was the only factor associated.

An earlier study on this topic was conducted from 1985 to 1990 including 704 patients with RA [9]. In this cohort, 27.6% of the patients reported that food affected RA symptoms, with 10.7% reporting worsening and 5.5% improving. Another study [10] was conducted between May and December 2015, including 217 patients with RA. Like the previous study, nearly one-quarter of the patients reported the influence of food on joint symptoms.

In the literature, some foods have an "inflammatory" effect and others an "anti-inflammatory" effect [10]. Foods most commonly reported to aggravate symptoms in patients with CIRD are red meat, sugary sodas, sugary desserts, coffee, alcohol, gluten, and dairy products; others are perceived as improving symptoms, especially fish, spinach, and red fruits [8,10]. In our study, red meat, fish, and legumes were the most frequently cited foods as aggravating symptoms, while the foods most frequently cited as improving the symptoms of the disease are honey, garlic, and olive oil. In a survey of 742 patients (290 patients with RA, 51 with juvenile RA, 87 ankylosing spondylitis, 51 with psoriatic arthritis, 65 with primary fibromyalgia, and 34 with osteoarthritis) [8], 33% of the patients with RA reported disease aggravation, pain, and joint swelling, after eating some foods such as meat, alcohol, coffee, sugar, and sweets.

These results are in accordance with those of a survey conducted by Felder et al. [11]. Sundström et al. [12] reported that seven patients with SpA had joint pain associated with a particular food, most often vegetables, fruits, or foods rich in flour.

Numerous prospective studies have evaluated the association between meat consumption and the risk of developing RA [13]. A recent prospective study confirmed that no association was observed between meat consumption and the risk of developing RA [14]. He et al. conducted a multicentre cross-sectional study, which showed that there were no significant differences between RA patients and healthy people in the consumption of red meat [15]. On the other hand, some studies have contradicted these results and shown a relationship between meat consumption and the risk of developing RA [16], but in these studies, this risk was increased by high meat consumption. In our study, 21.4% of the patients reported worsening of their symptoms after eating red meat.

Numerous studies have shown that high fish consumption has been associated with a reduced risk of developing RA [5]. A cross-sectional study by Tedeschi et al., including patients with RA, showed that patients who consumed fish twice a week had a lower DAS28-CRP than those who consumed it less than once a month [17]. It was also shown that an additional portion of fish per week was associated with a significant reduction in DAS28-CRP [17]. The protective effect of fish on the risk of RA may be due to the anti-inflammatory effect of omega-3 [18], but this relationship has not been clearly established. However, a prospective cohort study by Sparks et al. showed no protective effect of fish consumption on the risk of RA [19]. A previous meta-analysis showed an inverse association between fish consumption and RA [20]. In our study, fish intake worsened the patients' joint symptoms. There are several possible reasons for these contradictory results [21]. Among them, a high content of the trimethylamine N-oxide (TMAO) molecule in fish, associated with microbial dysbiosis, promotes RA [22].

Legumes have a potential antioxidant and anti-inflammatory effect, which has been demonstrated in the literature. These effects may be due to their richness in dietary fiber, starch, protein, and phenolic compounds [23]. However, in our study, 4.2% of the patients reported an aggravation after ingestion of legumes (beans++), which can be explained by the fact that our patients have a pejorative context towards legumes and that they confuse gout disease with rheumatic disease.

Extra virgin olive oil is characterized by its anti-inflammatory activity, which may explain its beneficial effects on RA. Furthermore, all studies that evaluated the influence of the Mediterranean diet on joint symptoms and inflammatory parameters, with extra virgin olive oil as the cornerstone, perceive that this type of diet is beneficial [23-25]. A recent systematic review on the influence of the Mediterranean diet in RA patients confirmed that the Mediterranean diet can be beneficial for RA patients in association with pharmacological treatment. In contrast, the effect of the Mediterranean diet on the prevention of RA has not been confirmed [26]. The anti-inflammatory potential of garlic and its constituents has been confirmed by scientific evidence [27]. In a randomized, double-blind, placebo-controlled study of 70 women with RA, they found that garlic supplementation in combination with disease-modifying therapy may have a clinical, biologic, and fatigue improvement in RA [28], which is consistent with the results of our study, in which 3.6% of the patients reported improvement after garlic consumption.

In our study, the factors associated with reporting that diet affects joint symptoms were the age of the patient and the type of rheumatic disease. Patients who reported an effect on symptoms were older than those who did not. This suggests that after a long course of rheumatic disease, patients search for other alternatives such as diet or dietary practices. Thus, the majority of these patients were RA, which may be explained by the fact that RA patients are often polymedicated, which may be associated with their use of non-drug treatment options such as certain diets.

However, Haugen et al. [8] report that the patients who reported that food affected RA symptoms were younger in contrast to our study, and other characteristics did not differ between those who reported that food affected RA and those who did not. Thus, in Tedeschi et al.'s study [10], the patients indicating that diet has an effect on joint symptoms were younger than those who did not.

Most of our patients were on methotrexate (cDMARDs), and half of these patients had good compliance. We thought that patients using disease-modifying therapy, including cDMARDs or bDMARDs, with good compliance are less likely to report that food affects their joint symptoms, due to the potent effect of these drugs. Poor compliance was not associated with reporting that diet or food affects joint symptoms.

On the other hand, other disease characteristics, including disease duration, disease activity, and functional impact, did not differ depending on whether patients reported that food affected their rheumatic disease. These results are compatible with a previous study [10].

Discussions about diet are reported by a minority of CIRD patients. In our study, 23.2% of the patients discuss the topic of diet with their rheumatologists; furthermore, the majority of patients have shown interest in the subject of diet and want to discuss it [29]. In a Swedish study, a large percentage of patients on a non-traditional mixed diet (low glycaemic, Mediterranean, vegetarian, or vegan) wanted to be a participant in the diet discussions. There is a well-known link between food and psychological well-being [30]; this may explain why patients on a non-traditional mixed diet may have a personal interest in the impact of diet on their disease. In our study, experience with a food that improves joint symptoms was the only factor associated with discussing the diet with a rheumatologist.

Limitations

The limitations of this study include its cross-sectional design, the small number of patients, and the use of a non-validated questionnaire. Another limitation of the study is that we used only 24 foods to assess their influence on joint symptoms.

## Conclusions

We found that nearly one-third of the patients in our study reported that diet had an impact on their joint symptoms, 25% reported that their symptoms had worsened, and 18% avoided certain foods because of worsening symptoms. The associated factors were principally age and type of rheumatic disease. Some foods, especially of the Mediterranean type, such as olive oil, appeared to improve joint symptoms, while other foods rich in protein, such as red meat, appeared to worsen joint symptoms. Future prospective randomized studies are needed to confirm the results of our study.

## Appendices

Questionnaire

1. Do you think that diet influences the activity and/or development of CIRD?

Yes                              No

2. Are you looking for information on the diet to adopt for your CIRD?

Yes                              No

If yes, by what means?

TV, radio                       Internet                        Doctor

3. Do you have experience with any foods that worsen your symptoms?

Yes                              No

If yes, which ones?

4. Do you have experience with any foods that improve your symptoms?

Yes                              No

If yes, which ones?

5. For each food listed above, please indicate whether it worsens, improves, or has no effect on your symptoms.

Garlic

Olive oil

Argan oil

Saffron

Honey

Black cumin

Fish

Ginger

Curcumin

Cloves

Fenugreek

Red meat

Eggs

Tea

Coffee

Tomatoes

Pomegranates

Soda

Chocolate

Milk

Yoghurt

Dates

Dried fruit

Legumes

6. Do you avoid any specific foods that you believe worsen your symptoms?

Yes                  No

If yes, which ones?

7. Are you adopting any dietary practices to improve your symptoms?

Yes                              No

If yes, which ones?

When?

Before the diagnosis of CIDR          When I was announced as CIRD          After I started my treatment

If not, why not?

I don't believe in it      Fear of side effects       Fear of interactions

8. Does fasting during Ramadan affect your symptoms?

Yes                  No

If yes, then:   improves                                 worsens

9. Do you fast to improve your symptoms?

Yes                  No

If so, what type of fasting?

10. Are you taking supplements to improve your symptoms?

Yes                  No

If yes, which ones?

Vitamin D         Vitamin C         Omega-3         Magnesium         Selenium         Others

If yes, do you feel an improvement?

11. Have you ever asked your rheumatologist for advice on diet?

Yes                              No

If not, why not?

Hasn't discussed the subject          Wouldn't be interested         Has no knowledge of the subject

12. Would you like to discuss this with your doctor?

Yes                              No
